# Three-Dimensional Printing and Supercritical Technologies for the Fabrication of Intricately Structured Aerogels Derived from the Alginate–Chitosan Polyelectrolyte Complex

**DOI:** 10.3390/gels11070477

**Published:** 2025-06-20

**Authors:** Natalia Menshutina, Andrey Abramov, Eldar Golubev, Pavel Tsygankov

**Affiliations:** Department of Chemical and Pharmaceutical Engineering, Mendeleev University of Chemical Technology of Russia, Miusskaya pl. 9, 125047 Moscow, Russia; chemcom@muctr.ru (N.M.); abramovandrey516@gmail.com (A.A.); eldgol01@gmail.com (E.G.)

**Keywords:** direct ink writing, heterophase system, supercritical process, supercritical sterilization, aerogel

## Abstract

Patient-specific scaffolds for tissue and organ regeneration are still limited by the difficulty of simultaneously shaping complex geometries, preserving hierarchical porosity, and guaranteeing sterility. Additive technologies represent a promising approach for addressing problems in tissue engineering, with the potential to develop personalized matrices for the growth of tissue and organ cells. The utilization of supercritical technologies, encompassing the processes of drying and sterilization within a supercritical fluid environment, has demonstrated significant opportunities for obtaining highly effective matrices for cell growth based on biocompatible materials. We present a comprehensive methodology for fabricating intricately structured, sterile aerogels based on alginate–chitosan polyelectrolyte complexes. The target three-dimensional macrostructure is achieved through (i) direct ink writing or (ii) heterophase printing, enabling the deposition of inks with diverse rheological profiles (viscosities ranging from 0.8 to 2500 Pa·s). A coupled supercritical carbon dioxide drying–sterilization regimen at 120 bar and 40 °C is employed to preserve the highly porous architecture of the printed constructs. The resulting aerogels exhibit 96 ± 2% porosity, a BET surface area of 108–238 m^2^ g^−1^, and complete sterility. The proposed integration of 3D printing and supercritical processing yields sterile, intricately structured aerogels with substantial potential for the fabrication of patient-specific scaffolds for tissue and organ regeneration.

## 1. Introduction

At the present time, bioengineering represents a field of great potential in the development of technologies for the creation of matrices that are conducive to the effective growth of tissue and organ cells. These matrices are to be based on biocompatible and biodegradable materials. The utilization of highly porous materials, such as aerogels, constitutes a salient domain of research. Aerogels are materials with low density (0.003–0.15 kg/m^3^), an open porous structure (up to 99%), and a high specific surface area (200–1000 m^2^/g) [1]. It is evident that aerogels possess a number of advantageous properties, which renders them a promising material for use in a variety of applications. These include the cultivation of cells [2], the fabrication of highly effective drug delivery systems [3], the development of energy storage devices [3], the utilization as catalysts [4,5], the function as sorbents [6,7], and the production of heat and sound insulating materials [8].

Aerogels derived from biopolymers, including alginate and chitosan, are non-toxic, biocompatible, and biodegradable, thus rendering them suitable for medical applications [9,10] and the pharmaceutical industry [11]. At present, aerogels based on biopolymers have already found their application as hemostatic agents [12,13], highly effective drug delivery systems [14], and in tissue engineering to obtain matrices for the growth of tissue and organ cells [15,16].

Of particular interest is a hybrid gel based on alginate and chitosan [17]. The formation of the hybrid structure is attributable to a chemical reaction between the alginate polyanion and the chitosan polycation, which results in the formation of the corresponding polyelectrolyte complex [18]. The formation of a stable gel based on the alginate–chitosan polyelectrolyte complex has the potential to yield fundamentally new materials, eliminate the use of aggressive cross-linking agents; enhance thermal, chemical, mechanical properties; and increase the stability of materials [19,20,21,22].

Nonetheless, fabricating aerogels with predefined geometries remains challenging owing to their intrinsic low density, high porosity, and consequent mechanical fragility. Currently, macroscopic shaping is achieved primarily by mold casting, a strategy that severely limits the attainable geometrical complexity. Furthermore, conventional techniques for constructing intricate architectures rarely deliver materials with precisely controlled porosity. These limitations can be overcome using additive manufacturing technology.

The application of additive manufacturing to aerogel fabrication enables the direct realization of intricate product geometries without the need for subsequent post-processing. This advancement markedly broadens the spectrum of functional material applications, wherein both a tailored internal structure and complex external geometry are critical.

The development of new materials with strictly defined properties for applications in medicine, pharmaceuticals, and biotechnology necessitates the identification of effective sterilization strategies [23,24,25]. At present, numerous sterilization methodologies have been approved by regulatory authorities and are regarded as particularly suitable for industrial implementation [26]. The methods employed include heat or steam sterilization [27], sterilization with ethylene oxide [28], and gamma irradiation [29]. Nonetheless, these methods exhibit notable shortcomings, foremost among them the detrimental impact of sterilizing agents on the structural integrity and physicochemical properties of the final products—a limitation especially pronounced in materials and devices composed of biopolymers, hydrophilic compounds, and heat-labile substances. Comprehensive sterilization strategies for such heat-sensitive, hydrophilic, or biologically derived products remain scarce. Consequently, there is an urgent need to devise mild sterilization protocols that preserve the structural and functional attributes of the finished materials.

The application of supercritical technologies shows significant promise for the sterilization of biomedical products. In supercritical carbon dioxide sterilization, operating parameters typically span 8–40 MPa in pressure and 35–90 °C in temperature. [30,31,32]. As demonstrated in the literature [31,32], experimental studies have shown that elevating pressure and temperature enhances microbial inactivation by increasing carbon dioxide solubility and accelerating its diffusion across the plasma membrane. Nevertheless, raising the pressure beyond 10 MPa has little additional effect on CO_2_ solubility in water [31]. At pressures near or above the critical point, increasing the temperature decreases fluid density and consequently diminishes the solvent capacity of carbon dioxide [33]. To enhance microbial inactivation, supplementary sterilants are employed in conjunction with supercritical carbon dioxide—hydrogen peroxide has proven the most efficacious additive, as demonstrated in [32].

This study details the fabrication of intricately structured aerogels derived from alginate–chitosan polyelectrolyte complexes. The target three-dimensional macrostructure is achieved via two additive-manufacturing techniques—direct gel extrusion and heterophase 3D printing—whose respective merits and limitations are experimentally delineated. The preservation of the porous structure and attainment of sterility are accomplished through a coupled supercritical carbon dioxide drying–sterilization protocol.

## 2. Results and Discussion

### 2.1. Rheological Study of Different “Ink” Composition

The dynamic viscosities of both sodium alginate–chitosan and partially cross-linked sodium alginate–chitosan systems were measured across all polymer concentrations examined. To evaluate the influence of polymer concentration on viscosity, measurements were conducted at a minimum shear rate of 0.01 s^−1^ (Table 1).

In the sodium alginate–chitosan system, increasing chitosan concentration elevates viscosity markedly—from 0.8 to 1800 Pa s. This rise reflects the higher solid-phase content, which requires greater shear stress to displace adjacent layers, thereby augmenting viscosity.

An analogous trend is observed when chitosan is incorporated into the partially cross-linked sodium alginate gel matrix: viscosity attains its maximum at the highest chitosan concentration. The high viscosity of the system has the potential to impede the implementation of layer-by-layer formation of products using the 3D printing process.

**Figure 1:**
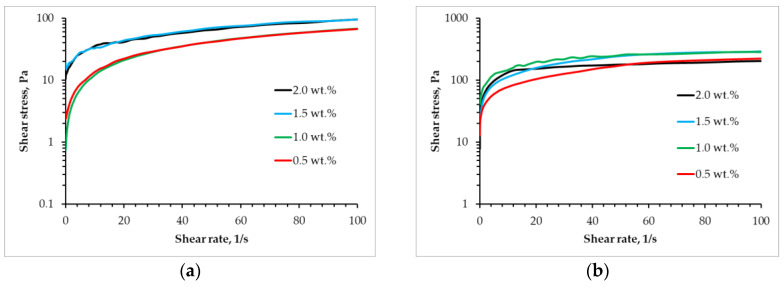
Flow curves for (**a**) alginate and (**b**) partly cross-linked alginate solution with different concentrations of chitosan.

One of the factors that must be considered when determining the possibility of using materials for the implementation of the three-dimensional printing process is the flow type [34]. In order to implement the three-dimensional printing process, it is necessary that the source material be characterized by a pseudo-plastic flow type, which is defined by a decrease in viscosity under the action of shear stresses. The present study investigates the rheological properties of gel materials of varying compositions, with a linear increase in shear rate from 0.01 to 100 s^−1^. The subsequent analysis of the obtained data resulted in the construction of flow curves (Figure 1).

The data reveal that, irrespective of formulation, the systems display pseudoplastic behavior, evidenced by rising stress with increasing shear rate. Furthermore, the degree of pseudoplasticity—that is, the deviation from Newtonian behavior—escalates proportionally with chitosan concentration in both sodium alginate–chitosan systems and partially cross-linked sodium alginate–chitosan systems.

A quantitative evaluation of thixotropic behavior was performed by increasing the shear rate from 0.01 s^−1^ to 100 s^−1^, followed by a decrement from 100 s^−1^ back to 0.01 s^−1^. Thixotropy curves were subsequently generated in shear stress–shear rate coordinates, as shown in the Supplementary Materials.

All formulations of the sodium alginate–chitosan and partially cross-linked sodium alginate–chitosan materials exhibited thixotropic hysteresis loops. Optimal thixotropic performance—i.e., maximal structural recovery—is attained when the loop area is minimized. The hysteresis loop areas were quantified for each composition (Table 2).

Sodium alginate–chitosan systems exhibit smaller hysteresis loop areas. Elevating the chitosan concentration enlarges the loop area, thereby retarding the recovery of the initial viscosity. Moreover, partial cross-linking of sodium alginate markedly attenuates thixotropic performance, as reflected by a pronounced increase in hysteresis loop area.

To assess the suitability of the formulated systems for 3D printing, their extrusion flow-onset points were evaluated. This parameter delineates the stress at which the material transitions from an elastically deformable solid to a viscous liquid under shear. The transition is marked by the yield point, defined as the intersection of the storage–modulus and loss-modulus curves.

**Figure 2:**
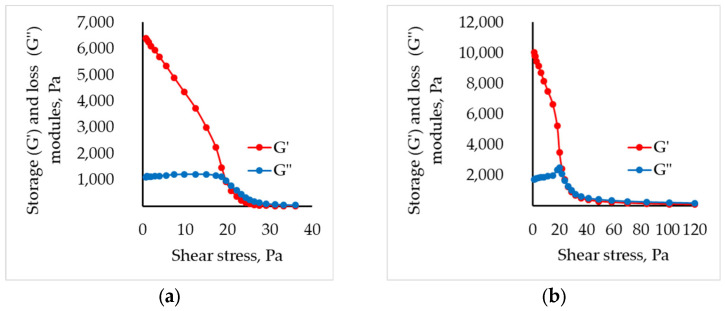
Storage (G′) and loss (G″) modulus for (**a**) alginate and (**b**) partly cross-linked alginate solution with 1.5 wt.% of chitosan.

The viscoelastic behavior of partially cross-linked sodium alginate solutions was evaluated at a constant angular frequency of 10 rad s^−1^. From these data, storage–modulus and loss–modulus curves were constructed for all formulations. As an example, Figure 2 depicts the dependencies at a chitosan concentration of 1.5 wt.%. (complete curves are provided in the Supplementary Materials).

The values of shear stresses required to initiate flow in the studied systems are presented in Table 3.

Experimental findings indicate that incorporating chitosan particles into sodium alginate solutions alters the flow-initiation point, whereas in partially cross-linked sodium alginate matrices the stress required to initiate flow declines as chitosan concentration increases. This behavior is plausibly attributable to the heightened solid-phase content and the presumably incomplete wettability of the chitosan particles, which induce structural instability and consequently reduce the onset stress.

It was determined that the addition of chitosan to a sodium alginate solution resulted in an increase in viscosity of the system, a decrease in thixotropic properties, and a shift in the flow onset point to a region of greater deformations. Conversely, when systems are formed based on partially cross-linked sodium alginate, the addition of chitosan results in an increase in viscosity and a decrease in thixotropic properties, analogous to materials based on non-cross-linked sodium alginate. However, it should be noted that for the “partially cross-linked sodium alginate–chitosan” systems, the flow onset point shifts to the region of smaller deformations with an increase in the chitosan concentration, which is presumably due to the incomplete wettability of chitosan particles. Subsequently, the resulting sodium alginate–chitosan and partially cross-linked sodium alginate–chitosan systems were studied as viscous “ink” for the implementation of extrusion 3D printing.

### 2.2. Three-Dimensional Printing Process

Conventional extrusion-based 3D printing deposits ink onto a planar substrate, necessitating rapid viscosity recovery and minimal post-extrusion spreading. Alternatively, objects can be printed within a heterophase medium, which supports the model during deposition and broadens the range of printable inks. In this study, gelatin microparticles constituted the heterophase matrix [35]. Printing parameters for each technique are detailed in Section 4.3.

Experimental investigations revealed that for the implementation of the 3D printing process with the formation of the objects by applying ink to the working surface, the necessary rheological characteristics are possessed by gel materials based on partially cross-linked sodium alginate with chitosan concentrations from 0.5 to 1.5 wt.%.

During printing with sodium alginate–chitosan formulations containing 0.5–1.5 wt.% chitosan, spreading of the material over the surface of the working area is observed due to insufficient viscosity and low stress values necessary to initiate the flow of gel materials. To use gel materials based on the sodium alginate–chitosan system with a chitosan concentration of up to 1.5 wt.% as “ink”, it is necessary to use the 3D printing process with the formation of the product in the volume of the heterophase system, which prevents the spreading of materials after the extrusion process.

At a chitosan concentration of 2 wt.%, the extruder nozzle consistently clogs as a result of the system’s elevated viscosity and high solid-phase content. Accordingly, the implementation of the 3D printing process using the developed gel material compositions is most affected by the solid phase (chitosan) concentration, the solution viscosity, and the flow onset point. The implementation of the 3D printing process, involving the application of ink to the working surface to form objects, imposes more stringent requirements on the ink. From a thixotropic standpoint, hysteresis loop areas of 2300–2900 are acceptable and ensure rapid viscosity recovery after deposition onto the build platform.

### 2.3. Integrated Supercritical Drying and Sterilization Processes

**Figure 3:**
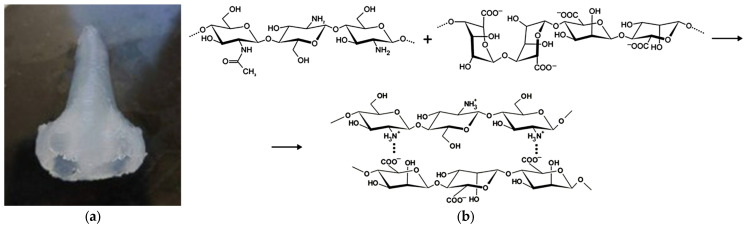
Three-dimensional-printed object based on “alginate–chitosan” solution with 1.5 wt.% concentration of chitosan after gelation process (**a**); mechanism of polyelectrolyte complex formation (**b**).

Complex-geometry objects printed within a heterophase medium were selected as model specimens for investigating combined supercritical drying and sterilization. Sodium alginate–chitosan systems served as the printing inks. To secure the final geometry, the printed objects were cryogenically fixed, and the frozen samples were immersed in 0.5 M hydrochloric acid at ambient temperatures for 24 h to dissolve the chitosan particles. During dissolution, a sodium alginate–chitosan polyelectrolyte complex forms via chemical coupling of alginate –COO^−^ groups with chitosan –NH_3_^+^ moieties, yielding a mechanically stable gel (Figure 3).

Subsequent to the completion of the gelation process, the solvent was gradually replaced with isopropyl alcohol in order to carry out the supercritical drying and sterilization process.

In accordance with the developed process flow chart and the design of the devices, following the completion of the supercritical drying process, the supercritical fluid sterilization process was carried out using an additional sterilizing agent, hydrogen peroxide.

The sterility testing procedure was performed in accordance with the Russian standard OFS 1.2.4.0003.15 “Sterility” [36] as well as internationally recognized standards, including USP <71> Sterility Tests, the European Pharmacopoeia (Ph. Eur.) 2.6.1 Sterility [37], and ISO 11737-1:2018 [38]. These standards ensure compliance with globally accepted methods for evaluating the effectiveness of sterilization processes.

The nutrient medium used for sterility testing was prepared by dissolving 31 g of dry thioglycollate medium powder in 1 L of distilled water. The solution was then boiled for 2 min and filtered. The pH of the medium was adjusted to 7.10 using hydrochloric acid (HCl). The medium was transferred to sterile flasks sealed with cotton-gauze stoppers and sterilized in a Stegler VK-18 autoclave at 121 °C and an overpressure of 1 atm for 15 min.

After completion of the supercritical carbon dioxide sterilization process at various mass flow rates, the materials were transferred aseptically to flasks containing the nutrient medium. All manipulations were performed inside a Biosan UVC/T-B-AR PCR box equipped with a UV recirculator. The flasks were then incubated at 37 °C for 14 days.

The results were visually recorded daily throughout the 14-day incubation period. Microbial growth was assessed by the appearance of turbidity, discrete spherical colonies, and other microscopic changes in the medium. The presence of microorganisms was confirmed by microscopic examination.

The experimental studies confirmed the sterility of the final materials under the selected process parameters.

### 2.4. Analytical Study of Hybryd Aerogel Based on Polyelectrolyte Complex

**Figure 4:**
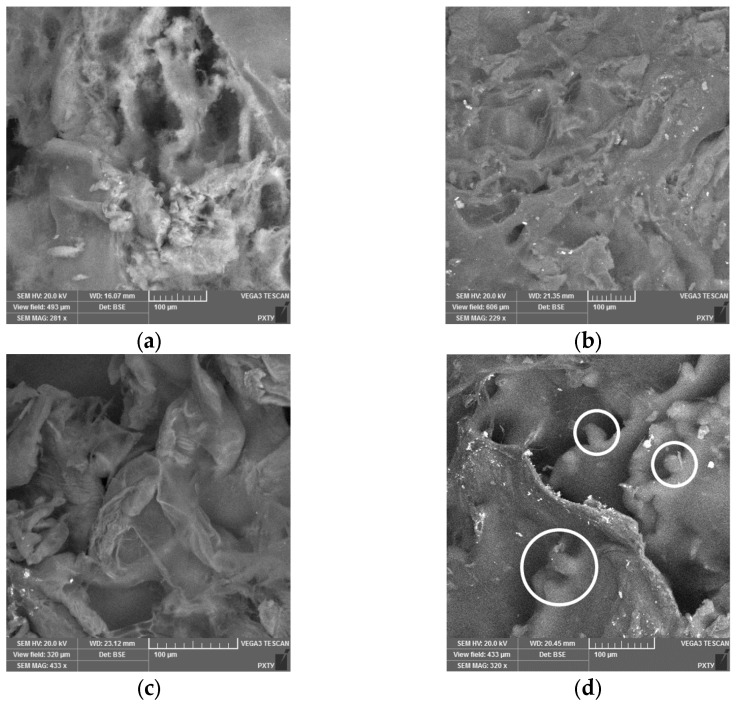
Images of the internal structure of aerogels based on a polyelectrolyte complex with different chitosan content (white circles presumably indicating chitosan particles coated with a polyelectrolyte shell): (**a**) 0.5 wt.%; (**b**) 1 wt.%; (**c**) 1.5 wt.%; (**d**) 2 wt.%.

As illustrated in Figure 4, the images depict aerogels derived from a polyelectrolyte complex of alginate and chitosan. The samples were obtained through the utilization of sodium alginate–chitosan ink, which was subsequently printed within the volume of the heterophase system. Notably, the sample exhibiting a chitosan concentration of 2 mass% was obtained without the employment of three-dimensional printing techniques. These images were obtained through the use of scanning electron microscopy (SEM).

As the concentration of chitosan increases, the resulting SEM images demonstrate that a more heterogeneous structure is formed. For the sample with a chitosan concentration of 2 wt.%, areas are identified that presumably represent chitosan particles coated with a polyelectrolyte shell. The formation of a heterogeneous structure is associated with the features of the process of obtaining the polyelectrolyte complex. The dissolution of chitosan particles is initiated upon contact with a hydrochloric acid solution.

**Figure 5:**
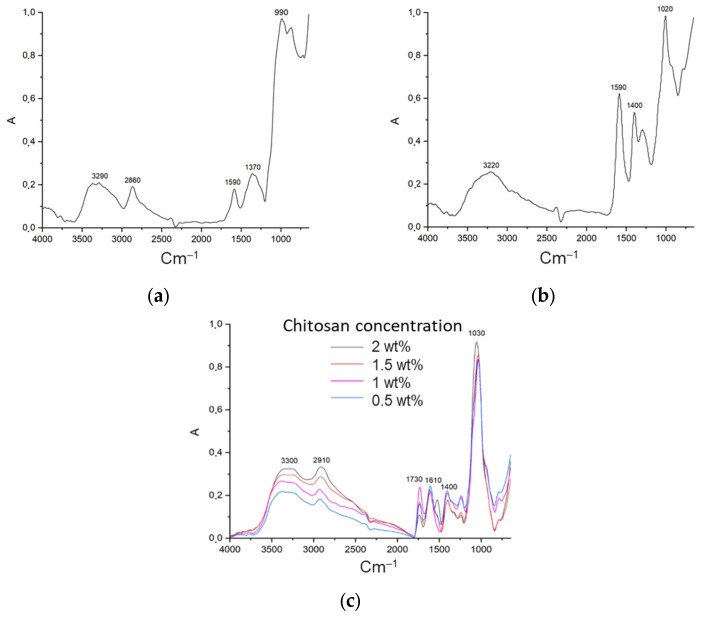
IR spectra of (**a**) chitosan; (**b**) sodium alginate; (**c**) aerogel based on polyelectrolyte complex.

In order to study the formation of the polyelectrolyte complex after the printing process, gelation and drying in a supercritical fluid medium, IR spectroscopy studies were carried out. As illustrated in Figure 5, the IR spectra of the initial sodium alginate and chitosan powders are presented. The spectrum of chitosan displays a broad band ranging from approximately 3000 to 3600 cm^−1^, indicative of the stretching vibrations of O–H and N–H bonds. The maximum at wave numbers of ∼2860 cm^−1^ corresponds to the vibrations of the C–H bonds of all hydrocarbon components. The peak observed at 1590 cm^−1^ is attributed to the overlap of the peaks belonging to the C-O and N-H groups.

The spectrum of sodium alginate is characterized by a broad band with a peak at ∼3220 cm^−1^, which corresponds to the O–H stretching vibrations. The presence of two peaks at ∼1590 cm^−1^ and ∼1400 cm^−1^ is attributed to the carboxyl group. At ∼1020 cm^−1^, C–O–C vibrations are observed.

The IR spectra of the obtained aerogels demonstrate the formation of a polyelectrolyte complex. The polyelectrolyte complex exhibits a more intense absorption band at ∼3300 cm^−1^, attributable to the formation of hydrogen bonds between the –OH and –NH_2_ groups in chitosan and the –C=O and –OH groups of sodium alginate. New peaks at approximately 1610 cm^−1^ and 1730 cm^−1^ were observed for all four alginate/chitosan ratios. The peak at 1610 cm^−1^ exhibits comparable intensity in all four samples. The peak at 1730 cm^−1^ corresponds to the asymmetric stretching of the –COO– groups, thus confirming the formation of a polyelectrolyte complex.

Table 4 presents the characteristics of aerogels based on the polyelectrolyte complex alginate–chitosan: true density (ρ_true_), apparent density (ρ_apparent_), porosity, BET-specific surface area (S_BET_), Barrett–Joyner–Halenda pore volume (V_BDH_).

An increase in the concentration of chitosan has been shown to result in an increase in true density, which in turn provides a reinforcing effect and a decrease in apparent density of the samples. It has been demonstrated that an increase in the concentration of chitosan results in a decrease in the specific surface area and the volume of mesopores. This phenomenon is associated with the formation of a non-uniform structure and the incomplete dissolution of chitosan.

Consequently, supercritical drying facilitates the production of hybrid aerogels derived from the polyelectrolyte complex sodium alginate–chitosan. The process of supercritical drying has been demonstrated to be an effective method for ensuring the preservation of the structural integrity of products obtained through the 3D printing process. The process of supercritical sterilization does not result in the degradation of the porous structure of the final products and allows the achievement of sterility.

## 3. Conclusions

This study investigates the fabrication of sterile complex-geometry aerogels based on sodium alginate–chitosan polyelectrolyte complexes via the integration of additive manufacturing and supercritical processing. The comprehensive rheological characterization of sodium alginate and partially cross-linked sodium alginate with the addition of chitosan was performed to select 3D printing technology and parameters. All gel compositions displayed pseudoplastic flow behavior: increasing shear rate elevated shear stress while reducing apparent viscosity. Elevating chitosan concentration markedly increased viscosity—from 0.8 to 1800 Pa s for sodium alginate solutions and from 1032 to 7970 Pa s for partially cross-linked sodium alginate. All formulations also exhibited thixotropy, characterized by partial structural breakdown under shear and recovery upon stress removal; thixotropic recovery declined as chitosan content increased in both matrix types.

Rheological analyses and printing trials indicated that inks based on partially cross-linked alginate containing 0.5–1.5 wt.% chitosan are optimal for direct gel extrusion. These formulations provide adequate viscosity, a pronounced yield stress, and rapid structural relaxation (hysteresis loop areas of ~2400–2700), preventing layer spreading and preserving geometric fidelity. Sodium alginate inks with comparable chitosan loadings require printing within a gelatin-based heterophase medium to suppress post-extrusion spreading.

For objects printed in the heterophane system, combined supercritical CO_2_ drying and sterilization were investigated. The analytical characterization confirmed preservation of a porous structure with high specific surface area (up to 238 m^2^/g) and large pore volume (up to 1.23 cm^3^/g). Increasing chitosan concentration to 2 wt.% degraded structural uniformity, as evidenced by scanning electron microscopy. Nevertheless, the integrated drying–sterilization protocol yielded sterile aerogels without compromising internal structure and macroscopic geometry.

In this work, an approach to the implementation of processes for obtaining highly porous matrices with complex geometry using additive and supercritical technologies is proposed. The potential for producing sterile aerogels based on the sodium alginate–chitosan polyelectrolyte complex using various additive manufacturing (direct gel printing and 3D printing using a heterophase system) and combined drying and sterilization processes in a supercritical carbon dioxide environment has been demonstrated.

## 4. Materials and Methods

### 4.1. Materials

Gelatin (CAS Number: 9000-70-8, Merck KGaA, Darmstadt, Germany) and anhydrous calcium chloride (RusChem, Moscow, Russia) were used to obtain a heterophase system. Alginic acid sodium salt (CAS Number: 9005-38-3, Sigma-Aldrich, St. Louis, MO, USA) and chitosan (CAS Number: 9005-38-3, Sigma-Aldrich, St. Louis, MO, USA) were used as ink precursors. Other materials, including distilled water, isopropanol, and hydrogen peroxide (36 wt.%), were purchased from RusChem (Moscow, Russia). Carbon dioxide with a purity of >99% was used for supercritical drying and sterilization.

### 4.2. Methods of “Ink” and Heterophase System Preparation

As demonstrated in [34], the optimal properties for the implementation of the printing process are exhibited by material compositions with a sodium alginate concentration of 2 wt.%. The concentration of chitosan in the materials for printing varied: 0, 0.5, 1, 1.5, and 2 wt.%. In this work, two schemes for obtaining viscous “ink” for the implementation of the three-dimensional printing process are proposed, depending on the selected technology.

#### 4.2.1. Three-Dimensional Printing Process Utilizing Heterophase System

In the implementation of 3D printing technology utilizing a heterophase system, the process of acquiring viscous “ink” derived from sodium alginate and chitosan entails the generation of a sodium alginate solution in distilled water, employing a rotor–stator homogenizer (IKA Ultra-Turrax T 25 digital, Staufen im Breisgau, Germany) at a rotor speed of 9000 rpm and a process duration of 2 min. Following the complete dissolution of sodium alginate in the resulting solution, the dispersion of the chitosan powder is carried out at a rotor speed of 12,000 rpm and a process time of 5 min. The values selected for the speed and time of the dispersion process are due to the necessity of achieving a uniform distribution of the chitosan powder throughout the volume of the solution.

#### 4.2.2. Direct Ink Writing (DIW)

In the implementation of the direct gel printing technology, a modified solution of partially cross-linked sodium alginate was utilized. Calcium chloride is dissolved in distilled water until a concentration of 0.2 wt.% is reached, using a magnetic stirrer. Chitosan powder is dispersed in the resulting solution by means of a rotor–stator homogenizer, with a rotor speed of 12,000 rpm and a process time of 5 min, until the required concentration is reached. Subsequently, sodium alginate powder with a 2 wt.% is dispersed.

#### 4.2.3. Heterophase System Preparation

To implement the 3D printing process using a modernized 3D printer construction, a method for obtaining a gelatin-based heterophase system was developed. A given amount of gelatin (4.5 wt.%) is dissolved in a solution of calcium chloride with a concentration of 11 mM at a temperature of 60 °C, followed by solidification at a temperature of 4 °C for 1 h. The resulting solution is dispersed in an 11 mM calcium chloride solution in a 3:7 volume ratio using a rotor–stator homogenizer (IKA Ultra-Turrax T 25 digital) at a rotor speed of 9000 rpm for 90 s. The resulting suspension is centrifuged (Sigma 2-16 PK, Osterode am Harz, Germany) at 4 °C to remove dissolved gelatin. The centrifugation process continues until a clear solution is obtained over the precipitate. Gelatin microparticles obtained as a result of repeated washing are used as a heterophase system for 3D printing.

### 4.3. Implementation of 3D Printing Process

**Figure 6:**
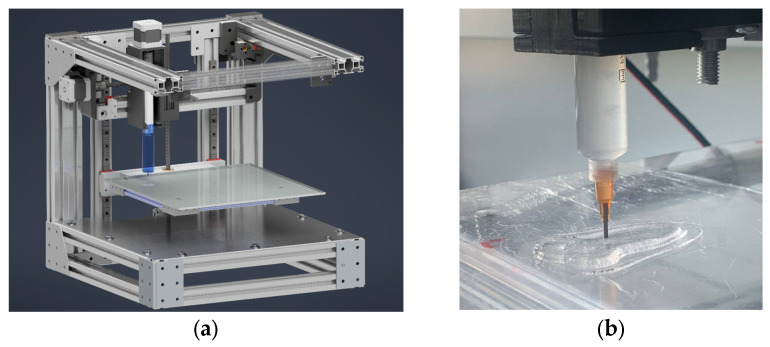
Construction of 3D printer to implement 3D printing process (**a**), example of implementation of 3D printing process using direct ink writing technology (**b**).

In this study, a 3D printer of our own design was utilized to implement the 3D printing process (Figure 6).

**Figure 7:**
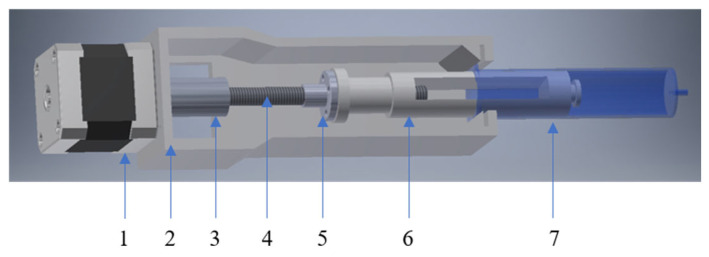
The assembly of the viscous “ink” extruder comprises the following components: 1—stepper motor; 2—housing; 3—coupling sleeve; 4—trapezoidal screw; 5—trapezoidal nut; 6—piston; 7—container for viscous “ink”.

The extruder of gel materials proposed in the work was used as a pressing device [34] (Figure 7).

In light of the disparate viscosity characteristics exhibited by the formulated ink compositions, the subsequent process parameters were employed in the implementation of direct gel printing technology: layer thickness, 1 mm; extruder nozzle speed, 5 mm/s; extruder piston speed, 0.1 mm/s; and printing temperature, 20 °C. The following parameters were utilized in the implementation of the 3D printing process using a heterophase system: layer thickness, 0.6 mm; extruder nozzle speed, 6 mm/s; extruder piston speed, 0.01 mm/s; and, printing temperature 20 °C.

### 4.4. Description of Integrated Supercritical Drying and Sterilization Processes

**Figure 8:**
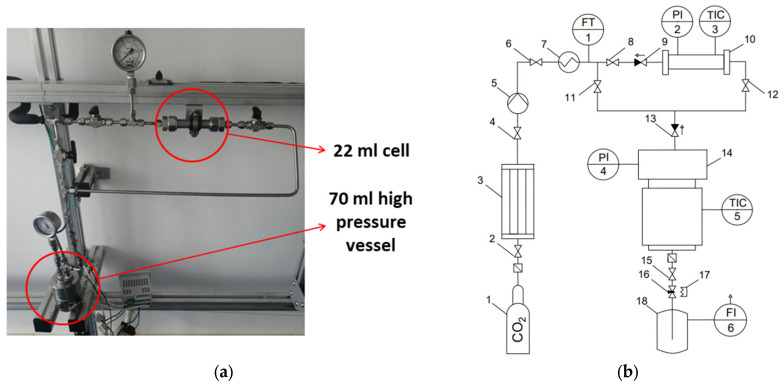
External appearance (**a**) and flowsheet (**b**) of the unit for combined processes of supercritical drying and sterilization: 1—cylinder with carbon dioxide; 3—condenser; 5—pump; 7—heat exchanger; 10—cell, 22 mL; 14—high-pressure apparatus, 70 mL; 17—heating element; 18—separator; 2, 4, 6, 8, 11, 12, 15—shut-off valves; 9, 13—check valves; 16—regulating valve; FT1—Coriolis flow meter; PI2, PI4—pressure sensors; TIC3, TIC5—temperature sensors and controllers; FI6—rotameter.

In order to implement the combined processes of drying and sterilization in a supercritical carbon dioxide environment, a laboratory setup with a high-pressure apparatus with a volume of 70 mL (for loading gel samples) and a cell of 22 mL (for the sterilizing agent) was utilized. The fundamental technological scheme and design of the apparatus are presented in Figure 8.

The valves 8, 11, and 12 ensure the switching of the drying and sterilization processes. At the initial moment of time, the necessary parameters for conducting combined processes are set in the high-pressure apparatus 14 and the intermediate tank 10. It has been established that the process of dissolving hydrogen peroxide in supercritical carbon dioxide occurs in intermediate tank 10 during the drying process, which occurs in flow mode. Subsequent to the conclusion of the drying process, valve 11 is closed, whilst valves 8 and 12 are opened in order to conduct the supercritical sterilization process with an additional sterilizing agent in a flow mode.

The supercritical drying process was conducted under the following parameters: temperature, 40 °C; pressure, 120–140 bar; carbon dioxide flow rate, 1000 g/h; and process time, 4–5 h. Upon the completion of the process, the pressure is released at a rate of 4 bar/min.

Based on previous studies, the process of the supercritical sterilization of aerogels based on the sodium alginate–chitosan polyelectrolyte complex was carried out at a mass flow rate of 500 g/h and a process time of 30 min in a flow mode through a cell with H_2_O_2_, a pressure of 120 bar and a temperature of 40 °C.

### 4.5. Analytical Study

The nitrogen adsorption–desorption isotherms were measured at −196 °C using a volumetric apparatus (ASAP 2020, Micromeritics, Norcross, GA, USA). The specific surface area was calculated using the BET method for isotherm linear range, and the total sorption mesopore volume was obtained at P/P0 = 0.95. Pore diameters were determined using the Barrett–Joyner–Halenda (BJH) algorithm. The BJH algorithm uses a modified Kelvin equation to link the removed adsorbed material from pores with the pore sizes.

Scanning electron microscopy imaging was performed on an SEM (JSM 6510 LV, JEOL, Akishima, Japan).

The determination of the viscosity characteristics was carried out on an Anton Paar MCT 302 rheometer with a plane–plane measuring unit type with a diameter of 25 mm and a temperature of 20 °C. In order to enhance the precision of the measurements and mitigate the impact of thixotropic properties on the outcomes, a relaxation time of 10 min was established for all studies.

## Figures and Tables

**Table 1:** Viscosity of sodium alginate–chitosan and partially cross-linked sodium alginate–chitosan systems on chitosan concentration.

Concentration of Chitosan, wt.%	0	0.5	1.0	1.5	2.0
Alginate	0.8 ± 0.1	82.2 ± 1.2	318.7 ± 9.6	1591.3 ± 79.6	1800.0 ± 47.2
Partly cross-linked alginate	1032.4 ± 12.5	1446.0 ± 21.1	1578.2 ± 24.9	1754.5 ± 35.1	7970.0 ± 184.1

**Table 2:** Areas of thixotropy hysteresis loops of the sodium alginate–chitosan and partially cross-linked sodium alginate–chitosan systems depending on the chitosan concentration.

Concentration of Chitosan, wt.%	0	0.5	1.0	1.5	2.0
Alginate	9.5	29.9	40.2	105.8	659.3
Partly cross-linked alginate	2374.2	2405.8	2416.1	2887.9	2967.8

**Table 3:** Values of shear stresses required to initiate the flow of the studied systems “sodium alginate–chitosan” and “partially cross-linked alginate–chitosan”.

Concentration of Chitosan, wt.%	0	0.5	1.0	1.5	2.0
Alginate	0	0.8 ± 0.1	1.8 ± 0.1	19.7 ± 0.1	52.8 ± 0.8
Partly cross-linked alginate	77.1 ± 2.1	42.8 ± 1.4	32.3 ± 0.6	26.3 ± 0.8	12.2 ± 0.1

**Table 4:** Characteristics of aerogels based on the polyelectrolyte complex sodium alginate–chitosan.

Concentration of Chitosan, wt.%	ρ_иcт_, g/cm^3^	ρ_кaж_, g/cm^3^	Porosity, %	S_BET_, m^2^/g	V_BDH_, cm^3^/g
0.5	1.684	0.090	95	238	1.23
1.0	1.853	0.099	95	199	0.59
1.5	1.884	0.076	96	153	0.64
2.0	1.982	0.062	97	108	0.37

## Data Availability

The original contributions presented in this study are included in the article/Supplementary Materials. Further inquiries can be directed to the corresponding author.

## Supplementary Materials

The following supporting information can be downloaded at: https://www.mdpi.com/article/10.3390/gels11070477/s1. Supplementary materials contain the results of complex rheological studies (Flow curves, Storage (G′) and loss (G″) modulus) of alginate and partly crosslink alginate solution with different concentrations of chitosan
