# Supplementary material for: Three-Dimensional Printing and Supercritical Technologies for the Fabrication of Intricately Structured Aerogels Derived from the Alginate–Chitosan Polyelectrolyte Complex
Source: Gels. 2025 Jun 20;11(7):477. doi: 10.3390/gels11070477 (PMC12294165; doi:10.3390/gels11070477)
Supplement: Supplementary file 1 [file gels-11-00477-s001.zip › gels-3671514-supplementary.pdf]

Concentration of chitosan, wt.%	Alginate	Partly crosslinked alginate
0,5	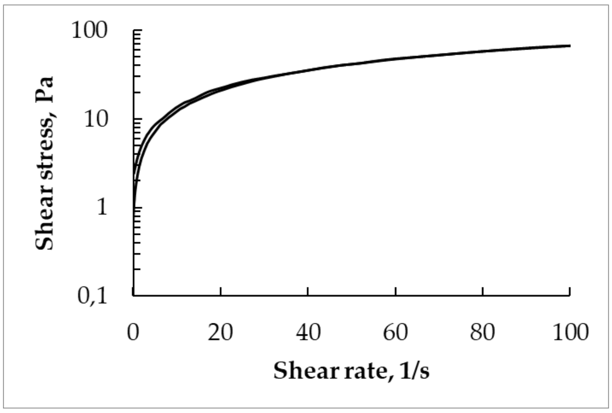	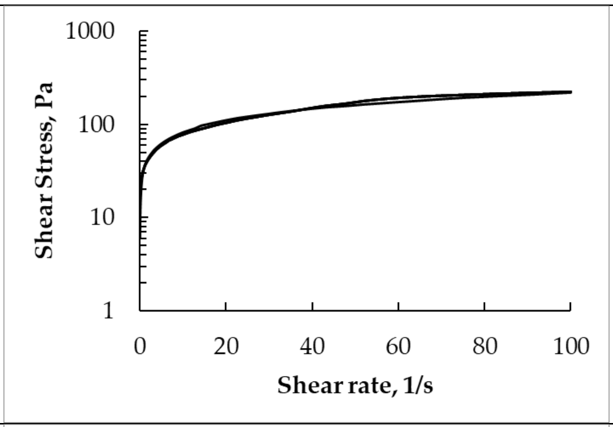
1,0	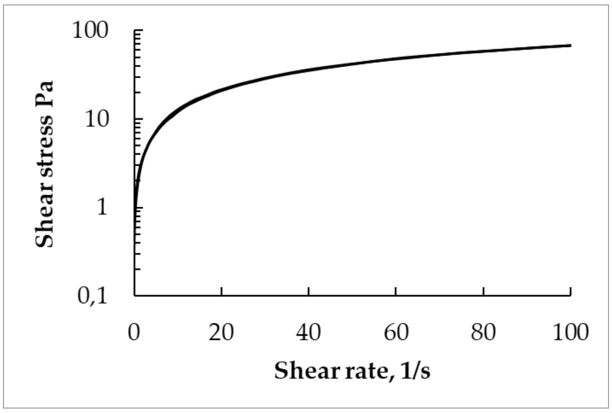	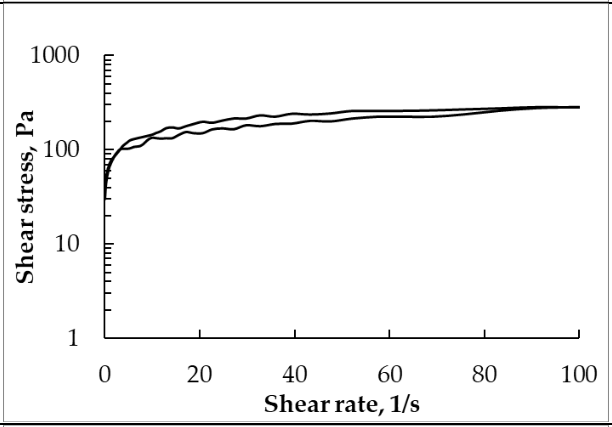
1,5	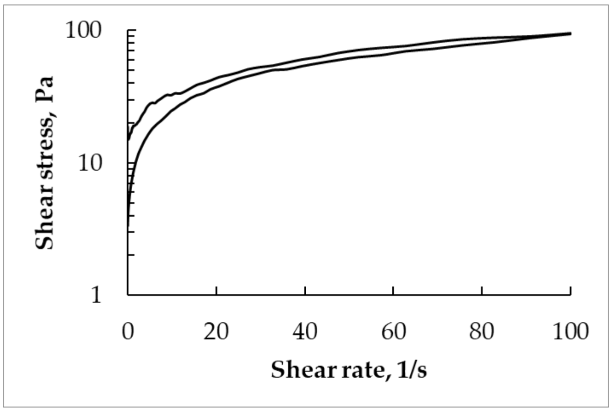	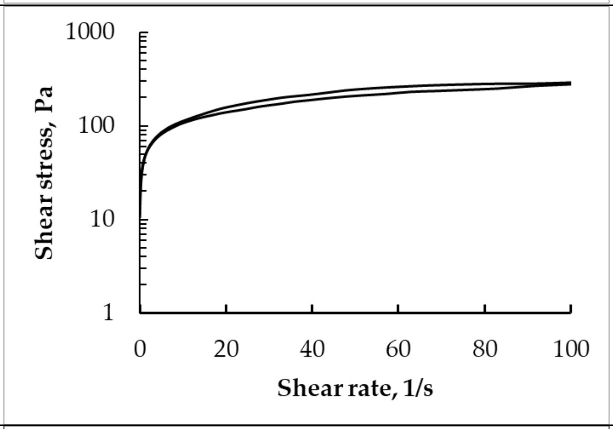
2,0	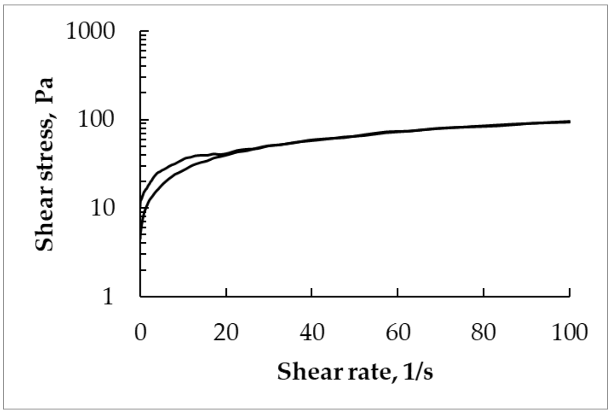	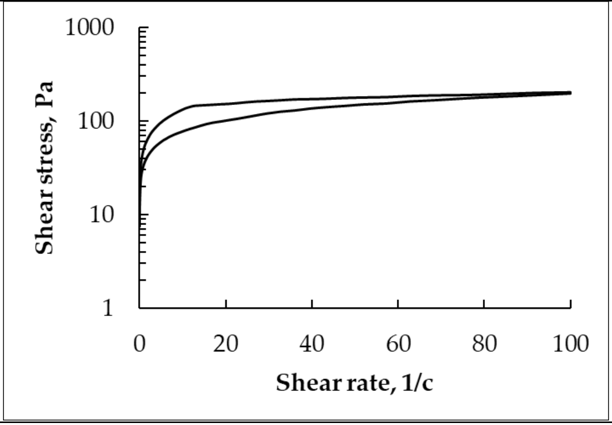

<table>
<tr><th>Concentration of chitosan, wt.%</th><th>Alginate</th><th>Partly crosslinked alginate</th></tr>
<tr><td>0</td><td>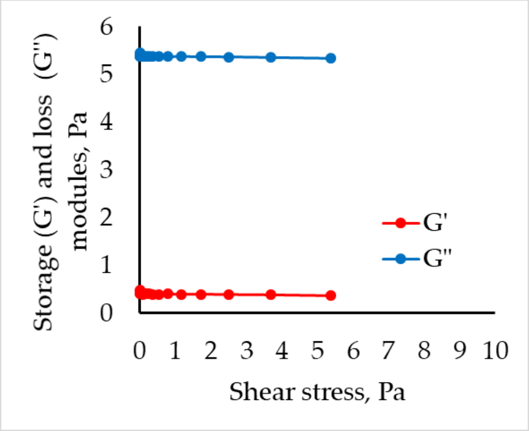
</td><td>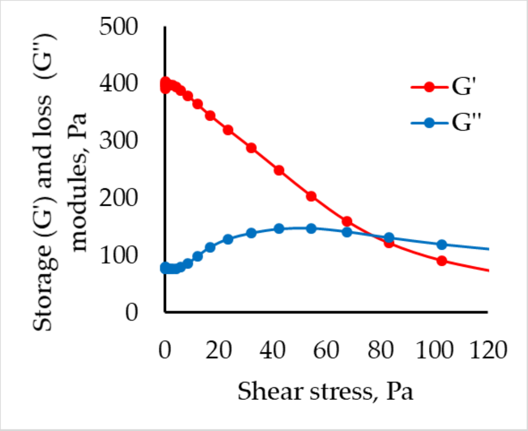
</td></tr>
<tr><td>0,5</td><td>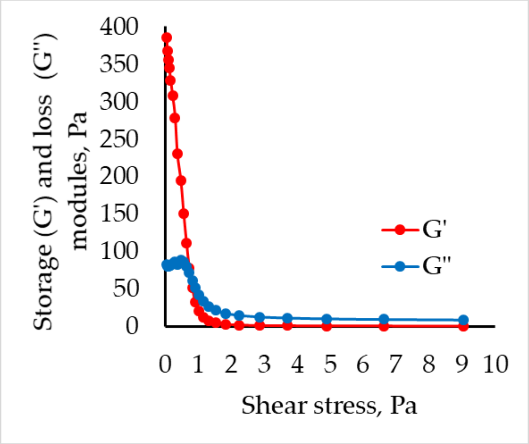
</td><td>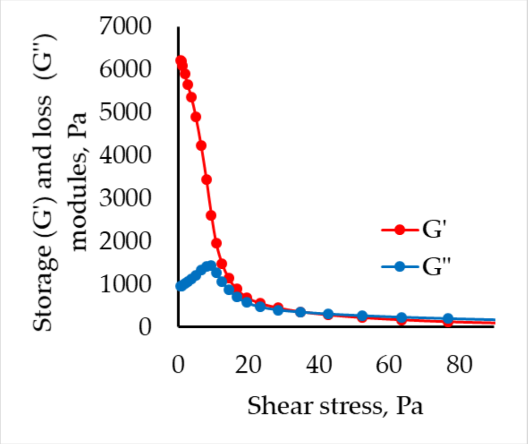
</td></tr>
<tr><td>1,0</td><td>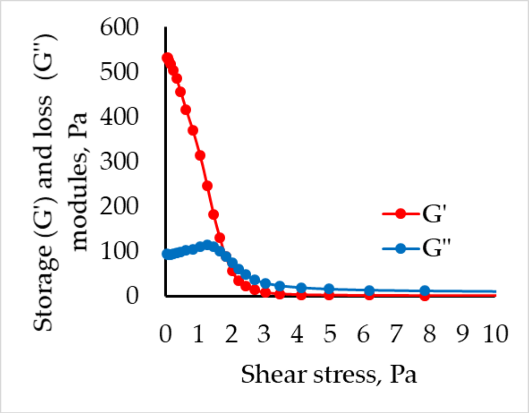
</td><td>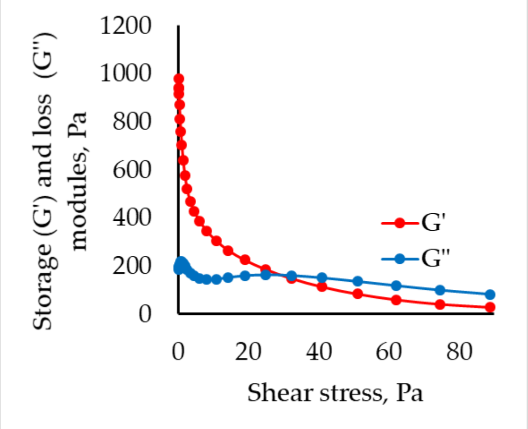
</td></tr>
<tr><td>1,5</td><td>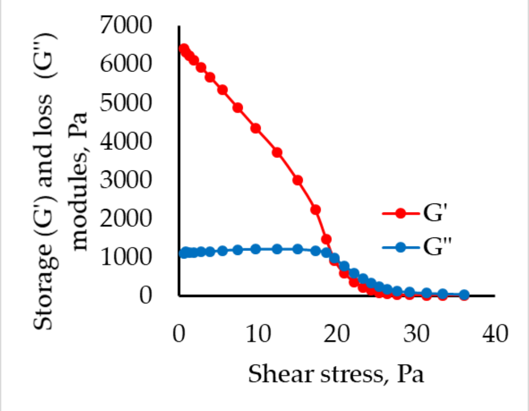
</td><td>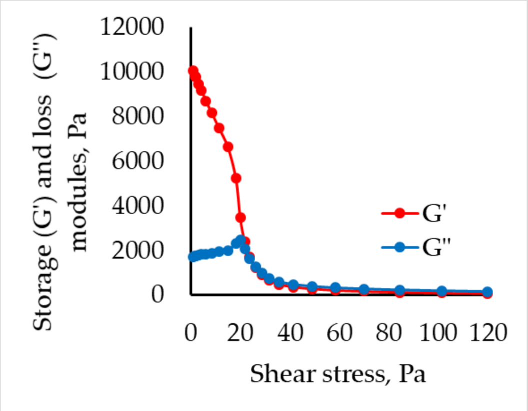
</td></tr>
</table>

2,0		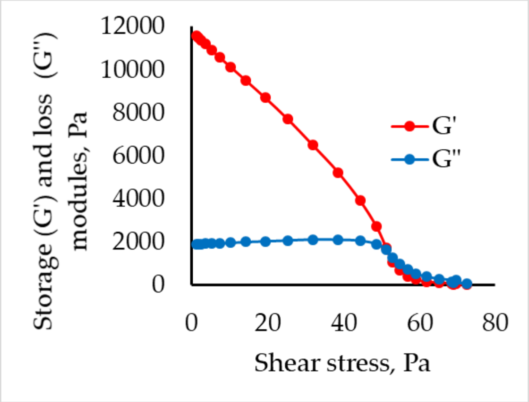12000
10000
8000
6000
4000
2000
0
0
20
40
60
80
Shear stress, Pa
Storage (G') and loss (G'') modules, Pa
G'
G''	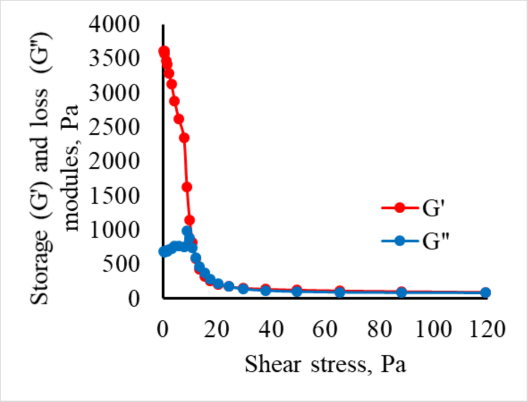4000
3500
3000
2500
2000
1500
1000
500
0
0
20
40
60
80
100
120
Shear stress, Pa
Storage (G') and loss (G'') modules, Pa
G'
G''
